# Relationships Between Circulating Lipids, Lipoproteins, and Lymphocyte Subsets in the Multi-Ethnic Study of Atherosclerosis

**DOI:** 10.5334/gh.1521

**Published:** 2026-02-03

**Authors:** Theodore M. DeConne, Colleen M. Sitlani, Joseph A. Delaney, Bruce M. Psaty, Margaret F. Doyle, James D. Otvos, Matthew J. Feinstein, Nels C. Olson

**Affiliations:** 1 Gerontology and Geriatric Medicine, Department of Internal Medicine, Wake Forest University School of Medicine, Winston-Salem, NC, US; 2 Department of Medicine, University of Washington, Seattle, WA, US; 3 Departments of Medicine and Epidemiology, University of Washington, Seattle, WA, US; 4 Cardiovascular Health Research Unit, Departments of Medicine, Epidemiology, and Health Systems and Population Health, University of Washington, Seattle, WA, US; 5 Department of Pathology and Laboratory Medicine, University of Vermont Larner College of Medicine, Burlington, VT, US; 6 Lipoprotein Metabolism Laboratory, Translational Vascular Medicine Branch, NHLBI, NIH, Bethesda, MD, US; 7 Division of Cardiology, Department of Medicine, Northwestern University Feinberg School of Medicine, Chicago, IL, US

**Keywords:** B-cells, T-cells, lipids, lipoproteins, epidemiology

## Abstract

**Background:**

Pre-clinical studies demonstrated lipids and lipoproteins influence T-cell phenotype. Several large cohort studies have also observed that plasma lipids and lipoproteins are associated with white blood cell and lymphocyte counts. However, there are little data on the relationships of lipids or lipoproteins with lymphocyte subsets in large, community-based, multi-ethnic cohorts.

**Objectives:**

The purpose of this study was to evaluate associations of plasma lipid and lipoprotein fractions with circulating lymphocyte subsets in participants of the Multi-Ethnic Study of Atherosclerosis (MESA).

**Methods:**

MESA recruited 6,814 adults (aged 45–84 years) free of clinical cardiovascular disease at the baseline exam between 2000–2002. This study included 1,735 participants (49% male, 36% White) with lipoprotein and immune cell phenotyping data at baseline. Multivariable linear regression models evaluated associations between lipoprotein concentration (analyzed per 1-standard deviation (SD) increment) and lymphocyte subsets.

**Results:**

Following correction for multiple hypothesis testing (p < 0.0006), higher high-density lipoprotein (HDL)-cholesterol was associated with higher proportions of memory B-cells, while HDL-lipoprotein concentration was associated with lower pan B-cells. In analyses not corrected for multiple hypothesis testing (p < 0.05), higher concentrations of total-cholesterol, low-density lipoprotein (LDL) cholesterol and LDL-lipoproteins, triglycerides and triglyceride-rich lipoproteins were associated with higher proportions of several T-cell subsets associated with inflammation and senescence. Conversely, a higher concentration of HDL-lipoproteins were associated with lower proportions of senescence-associated T-cells.

**Conclusions:**

These results indicate plasma lipids and lipoproteins may play a role in influencing circulating immune cells. If confirmed in longitudinal studies, these findings may have implications for the development of therapeutics targeting inflammation in patients with elevated lipids.

## Graphical Abstract

**Figure:**
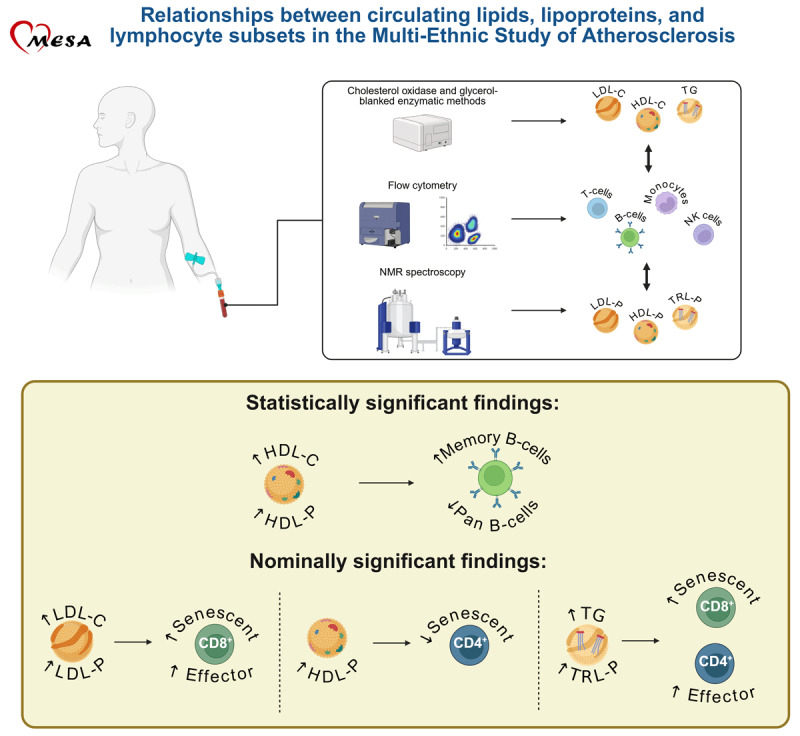
Created in BioRender. Deconne, T. (2025) https://BioRender.com/rkk95f3. Abbreviations: LDL-C, low-density lipoprotein-cholesterol; HDL-C, high-density lipoprotein-cholesterol; TG, triglycerides; NK, natural killer; LDL-P, low-density lipoprotein particles; HDL-P, high-density lipoprotein-particles; and TRL-P, triglyceride-rich lipoprotein-particles.

## Introduction

Sustained low-grade inflammation contributes to the development of multiple age-related diseases caused, in part, by the aging immune system (1,2,3). Adaptive immune cells, including T-lymphocytes (T-cells), are increasingly thought to contribute to inflammation and the development of age-related diseases (3,4,5). Concurrent with inflammation, aging is commonly associated with an increase in serum cholesterol, cholesterol-carrying lipoprotein particles (6,7,8), and intracellular T-cell cholesterol concentrations (9). Beyond playing essential roles in cell membrane integrity and lipid transport, cholesterol and lipoproteins are involved in immune regulation (10,11,12,13,14,15,16,17,18,19). Specifically, T-cell proliferation, differentiation, function, metabolism, and intracellular signaling can be regulated by the interaction of lipoproteins with the T-cell receptor or intracellular cholesterol accumulation and metabolism (14,15,16,17). It is now appreciated that low-density lipoprotein (LDL), oxidized LDL, and apolipoprotein B100 (apoB100) are presented to T-cells and induce activation (17). LDL can interact with the LDL receptor or the T-cell receptor on the T-cell membrane to influence cellular phenotype and function, independent of intracellular cholesterol (20,21). Evidence also indicates that cholesterol can bind the T-cell receptor and affect the interaction with antigens (22).

T-cell regulation by intracellular cholesterol or lipids is, in part, due to their localization. For example, T-cell membrane cholesterol, which accounts for the greatest proportion of total cellular cholesterol, is necessary for T-cell proliferation, CD4+ and CD8+ effector function, and memory T-cell differentiation (14,15,16,17). Additionally, lipid catabolism in the mitochondria also affects the formation and function of memory T-cells, while lipid droplet and membrane cholesterol accumulation may influence T-cell senescence (14,16). The accumulation of lipids in other organelles, such as the endoplasmic reticulum is also important. Lipid droplets formed in the endoplasmic reticulum (23), and cholesterol accumulation, can induce T-cell exhaustion by promoting endoplasmic reticulum stress (16,17,24). Many other studies have consistently shown that intracellular cholesterol influences inflammatory T-cell phenotype and function (25,26,27,28,29,30,31,32,33,34).

In addition to intracellular cholesterol, plasma cholesterol concentrations have also been linked to T-cell differentiation. For example, hypercholesterolemia in mice induced T regulatory (TREG) and Th17 T-cell expansion (35), promoted a shift from Th1 to Th2 T-cells (36), and from TREG towards pro-inflammatory Th1 and T-follicular helper T-cells (37). Observations from epidemiological studies also support a role of plasma lipids in regulating T-cell differentiation. A prior study from the Multi-Ethnic Study of Atherosclerosis (MESA) observed higher triglyceride concentrations, but not high-density lipoprotein cholesterol (HDL-C) or low-density lipoprotein cholesterol (LDL-C), were associated with a higher circulating lymphocyte count (38). Cross-sectional associations between lipids or lipoprotein subclasses with lymphocytes were also observed in the Copenhagen General Population Study, UK Biobank, and LifeLines DEEP cohort (39,40,41). Additionally, data from the MESA, National Health and Nutrition Examination Survey (NHANES), and UK Biobank suggested that sex might influence the association between plasma lipids and lymphocytes (38,39,42).

One observational study identified associations between lipoproteins and specific T-cell subsets (43). However, relationships between lipids and lipoproteins and specific immune cell subsets in large, community-based, multi-ethnic human cohorts are poorly understood. Further understanding of these relationships could inform the development of novel, patient-specific therapeutics to target inflammation in diverse populations with cardio-metabolic diseases and elevated lipids. Therefore, our study aimed to identify associations of plasma lipids and lipoproteins with a large panel of circulating lymphocyte subsets among 1,735 men and women from four race/ethnic groups who were not using lipid-lowering medications. We also aimed to leverage the diverse MESA cohort to further explore potential differences in the relationships by sex and race/ethnicity. We hypothesized that plasma lipid and lipoprotein concentrations would be associated with variation in circulating T-cell frequencies, which may have implications for inflammatory and cardiometabolic diseases.

## Materials and Methods

### MESA cohort

MESA is a prospective, community-based cohort study designed to study risk factors for subclinical atherosclerosis, atherosclerosis progression, and clinical atherosclerotic cardiovascular disease [CVD (44)]. At the baseline exam in 2000–2002, MESA recruited 6,814 men and women (aged 45–84 years) from six communities across the US (Chicago, IL; Los Angeles, CA; Baltimore, MD; St Paul, MN; New York, NY; and Forsyth County, NC). The cohort was 38% White, 28% African American, 22% Hispanic, and 12% Chinese. Those with clinical CVD or undergoing cancer treatment were excluded from study enrollment. Follow-up exams occurred in 2002–2004, 2004–2005, 2005–2007, 2010–2011, 2016–2018, and 2022–2024. At MESA Field Center clinics, participants underwent assessment for CVD risk factors and provided fasting blood samples. Standardized questionnaires were used to obtain demographic and lifestyle CVD risk factor information. Medication use was assessed by a standardized medication inventory.

The current study leveraged baseline (2000–2002) data from a nested case-cohort study designed to evaluate relationships of immune cell subsets with incident coronary heart disease (CHD) and heart failure (HF). The case-cohort study sampled all participants with incident CHD and HF and a random cohort (45,46). We leveraged this data for a secondary cross-sectional analysis of immune cell relationships with nuclear magnetic resonance (NMR) lipoprotein particle data available from the baseline Exam.

Covariates of interest in the current study included age, sex, race/ethnicity, body mass index (BMI), smoking status, lipid lowering medication use, systolic blood pressure (SBP), hypertension medication use, diabetes, estimated glomerular filtration rate (eGFR), and cytomegalovirus (CMV) IgG antibodies. BMI was calculated as the ratio of weight to height squared (kg/m^2^). Diabetes was defined as a fasting blood glucose ≥126 mg/dL or use of insulin or hypoglycemic medications. Serum creatinine was used in the CKD-EPI equation to calculate eGFR. IgG antibodies to CMV were measured by immunoassay (Diamedix Corp., Miami Lakes, FL; coefficient of variation 5.1–6.8%).

### Study approval

Written informed consent was obtained from all participants prior to their participation, and all procedures received approval from the Institutional Review Board for human subjects research (University of Washington, #00006878) and were performed in accordance with the principles stated in the Declaration of Helsinki. The authors confirm that patient consent is not applicable to this article. This is a retrospective analysis using de-identified data; therefore, additional consent was not needed for this study.

### Cellular phenotyping

Cellular phenotyping was performed among a case-cohort sample of 2,111 participants. All participants who had cryopreserved cells available at the baseline exam and incident CHD and HF, and a random cohort, selected by simple random sampling, were included. Detailed laboratory and flow cytometry methods including reagents, sample handling, and flow cytometry gating strategies are published in detail (45,46,47). Briefly, 8-mL citrate CPT tubes drawn at the MESA baseline exam (2000–2002) were used to isolate and cryopreserve peripheral blood mononuclear cells (PBMCs) at –135°C in media containing 90% fetal bovine serum and 10% dimethyl sulfoxide. PBMCs were thawed for 15 minutes at 37°C, washed, filtered and treated with a fixable viability stain using a standardized protocol (45,46). Cellular phenotyping of innate lymphocytes (i.e., natural killer, γδ T), CD14+ monocyte subsets, CD19+ B-cell subsets, and naive, memory, differentiated/effector or senescent (e.g., CD28– and CD57+), TEMRA CD4+ and CD8+ T-cell subsets, and regulatory (CD4+CD25+CD127–) T-cells was performed by cell-surface labelling. Intracellular cytokine staining was performed to phenotype CD4+ and CD8+ T-cells that produce interferon-γ (Th1, Tc1), IL-4 (Th2, Tc2), and IL-17A (Th17, Tc17). Cells were stimulated with phorbol myristic acetate and ionomycin in the presence of Brefeldin A to inhibit cytokine secretion (48). For intracellular cytokine staining, T-cells were first stained for CD4 and CD8, washed, fixed, and permeabilized to determine intracellular production of IFN-γ, IL-4, and IL-17A, using a standardized protocol (45,46). The cellular markers and their descriptions are included in Supplementary Table 1. Cellular phenotyping was assessed using an MQ10 flow cytometer and MACS Quantify software (Miltenyi Biotec). Each cell phenotype was expressed as a proportion of the parent population. The number of cellular phenotypes measured per participant varied due to occasional poor sample quality or technical errors. Missing immune cell data occurred at random and were not related to participant characteristics.

### Measurement of blood lipids and lipoprotein particles

Total-cholesterol, HDL-C, and triglycerides were measured in all participants at the baseline exam in fasting plasma samples. Total-cholesterol and HDL-C were measured by cholesterol oxidase method (Roche Diagnostic, Indianapolis, IN). Triglyceride concentrations were measured with the Triglyceride GB reagent using a glycerol-blanked enzymatic method (Roche Diagnostics). The Friedewald equation was used to calculate LDL-C in samples with triglycerides below 400 mg/dL.

Plasma lipoprotein particle concentrations were measured in all participants at the baseline exam by NMR spectroscopy at LipoScience, Inc. (now Labcorp, Morrisville, NC) using the NMR Profiler platform with the LP4 algorithm, as previously described (49). The assay quantifies the concentrations of different-size triglyceride-rich lipoprotein particles (TRL-P), low-density lipoprotein particles (LDL-P), and high-density lipoprotein particles (HDL-P) (50). Lipoprotein particle subclasses were differentiated by particle size (nm diameter). TRL-P subclasses were defined as very large (90–240 nm), large (50–89 nm), medium (37–49 nm), small (30–36 nm), and very small (24–29 nm). LDL-P subclasses were defined as large (21.5–23 nm), medium (20.5–21.4 nm), and small (19–20.4 nm); and HDL-P subclasses as large (9.6–13 nm), medium (8.1–9.5 nm), and small (7.4–8.0 nm) HDL-P.

### Statistical analysis

2,111 participants had immune cell phenotype data, three of whom did not have information on lipid-lowering medication use. Of the remaining 2,108 participants, 369 participants were excluded because they were on lipid-lowering medications and four participants were excluded because they did not have NMR spectroscopy data. This resulted in a final sample size of 1,735 participants. Linear regression models, weighted by inverse probability of inclusion in the immune cell case-cohort study, were used to evaluate associations between lipoprotein concentrations (independent variable; analyzed per 1-standard deviation (SD) increment) and immune cell subsets (dependent variable). The models were fit using the ‘lm’ function in R, which uses weight least squares. Robust standard error estimates were used to test hypotheses and obtain confidence intervals.

Models were adjusted for age, sex, race/ethnicity, MESA site, cell phenotyping analytical batch, BMI, smoking, hypertensive medications, SBP, diabetes, eGFR, and CMV. Robust standard error estimates were used to account for sample weighting. Statistical analyses were conducted in R version 4.1.2. Given the correlated nature of both the lipoprotein/cholesterol data and the immune cell data, we accounted for multiple testing by using a Bonferroni correction that incorporated the estimated number of independent lipoprotein/cholesterol measures [4] and the estimated number of independent immune cell measures [21], yielding a significance level of alpha < 0.0006 (0.05/21/4). For each data type, the estimated number of independent measures is the number of principal components that account for 99% of the variance in that data type (51). We interpreted p-values between 0.0006 and 0.05 as borderline or ‘nominally’ significant for the purposes of hypothesis generation.

We assessed lipid/lipoprotein-by-sex and lipid/lipoprotein-by-race/ethnicity interaction terms and defined p-interaction < 0.05 as significant. Stratified analyses were performed if there was a significant interaction. However, as the number of Chinese American participants was too small to make inferences about an interaction by race/ethnic group, these participants were excluded from these analyses.

### Sex as a biological variable

Both men and women were included in the analysis. Sex as a biological variable was assessed by testing for interaction effects of sex, which was provided by participant self-report, in each of the models.

## Results

### Participant characteristics

The demographic characteristics at the baseline examination of participants included in the analyses are described in Table 1. The average participant age was 62 ± 11 years; 51% of participants were women and 36% were White. On average, participants had total cholesterol and HDL-C within the normal range, and above optimal LDL-C. Most participants were not on hypertensive medications (65%) and did not have diabetes (87%).

**Table 1:** Participant descriptive characteristics.


	MEAN (SD), COUNT (%), OR MEDIAN (IQR)

Total n	1,735

Age, years	62 (11)

Male, %	852 (49%)

**Race/Ethnicity**	

Black, %	480 (28%)

Chinese, %	240 (14%)

Hispanic, %	398 (23%)

White, %	621 (36%)

**CVD Risk Factors**	

BMI, kg/m^2^	28 (5)

SBP, mmHg	128 (22)

Hypertension medications	616 (35%)

Diabetes	227 (13%)

eGFR, mL/min/1.73 m^2^	78 (17)

Smoking never, %	862 (50%)

Smoking former, %	632 (36%)

Smoking current, %	241 (14%)

**Blood Lipids (Median (IQR))**	

Total cholesterol, mg/dL	193 (172, 216)

LDL-C, mg/dL	118 (99, 138)

HDL-C, mg/dL	48 (40, 58)

Triglycerides, mg/dL	111 (78, 166)

**Blood Lipoproteins (Median (IQR))**	

Total LDL-P, nmol/L	1642 (1408, 1918)

Large LDL-P, nmol/L	127 (43, 286)

Medium LDL-P, nmol/L	209 (45, 462)

Small LDL-P, nmol/L	1134 (812, 1522)

Total HDL-P, μmol/L	21 (19, 23)

Large HDL-P, μmol/L	2 (2, 4)

Medium HDL-P, μmol/L	3 (1, 5)

Small HDL-P, μmol/L	15 (13, 17)

Total TRL-P, nmol/L	168 (128, 212)

Small TRL-P, nmol/L	0.1 (0.1, 0.2)

Very Small TRL-P, nmol/L	3 (1, 8)

Medium TRL-P, nmol/L	21 (11, 35)

Large TRL-P, nmol/L	86 (49, 123)

Very Large TRL-P, nmol/L	44 (22, 75)


Participant characteristics at the baseline MESA examination are presented. Data are presented as mean (SD), count (%), or median (interquartile range (IQR)). Abbreviations: CVD, cardiovascular disease; BMI, body mass index; SBP, systolic blood pressure; eGFR, estimated glomerular filtration rate; LDL-C, low-density lipoprotein-cholesterol; HDL-C, high-density lipoprotein-cholesterol; LDL-P, LDL particles; HDL-P, HDL particles; and TRL-P, triglyceride rich lipoprotein particles.

### Blood lipids

**Figure 1:**
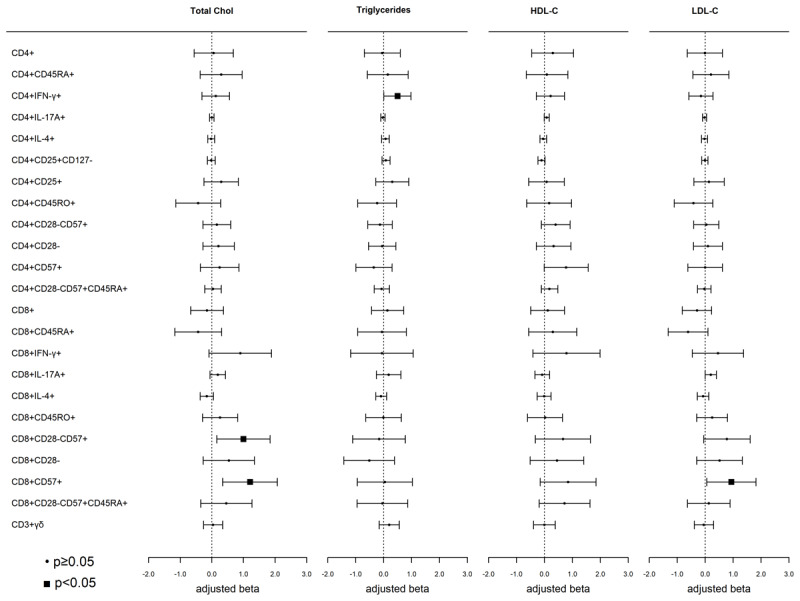
**Associations of blood lipids with T-cell subsets:** Weighted linear regression models were used with blood lipids as the independent variable and T-cell subsets as the dependent variable. Models were adjusted for age, sex, race/ethnicity, MESA site, cell phenotyping analytical batch, BMI, smoking, hypertensive medications, SBP, diabetes, eGFR, and CMV. Each model represents a single exposure. Results are presented as beta (95% CI), p-value. Abbreviations: Chol, cholesterol; HDL-C, high-density lipoprotein-cholesterol; and LDL-C, low-density lipoprotein-cholesterol.

Total cholesterol, LDL-C, HDL-C, and triglycerides were not significantly associated with any circulating T-cell subsets after adjusting for multiple comparisons (all p > 0.0006, Figure 1). However, we did observe nominal associations (p < 0.05) between total cholesterol and HDL-C with senescent-associated T-cell subsets. Higher (1–SD) total cholesterol concentration was nominally associated with higher proportions of CD8+CD28–CD57+ and CD8+CD57+ T-cells, and higher LDL-C was nominally associated with a higher proportion of CD8+CD57+ T-cells (Figure 1). Higher triglycerides were associated with higher Th1 (CD4+IFN-γ+) T-cells.

### Low-density lipoprotein particles (LDL-P)

**Figure 2:**
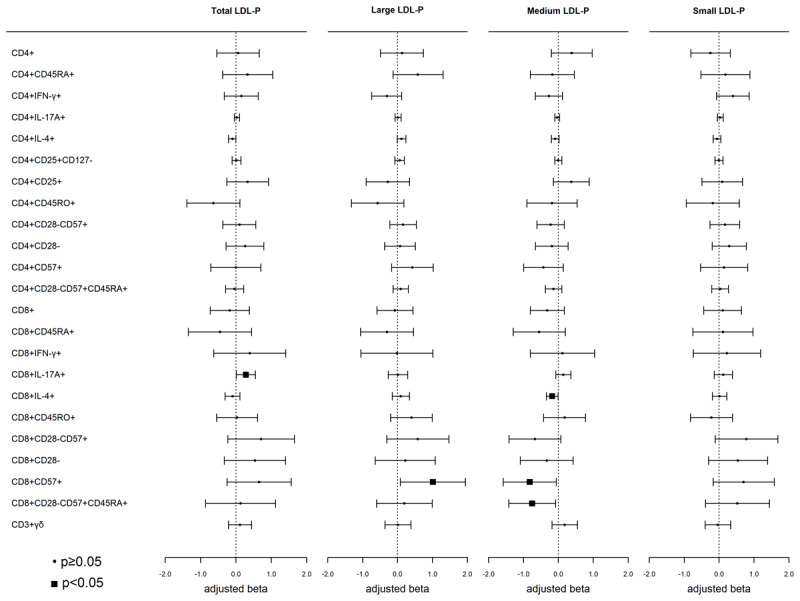
**Associations of low-density lipoprotein particle (LDL-P) fractions and T-cell subsets:** Weighted linear regression models were used with LDL-P as the independent variable and T-cell subsets as the dependent variable. Models were adjusted for age, sex, race/ethnicity, MESA site, cell phenotyping analytical batch, BMI, smoking, hypertensive medications, SBP, diabetes, eGFR, and CMV. Each model represents a single exposure. Results are presented as beta (95% CI), p-value.

There were no statistically significant relationships between LDL-P sub-fractions and any T-cell subset (all p > 0.0006) (Figure 2). Nominal associations, however, were observed with effector and senescent-associated T-cell subsets. 1–SD higher total LDL-P was associated with a higher percentage of Tc17 (CD8+IL-17 A+) T-cells. Higher large LDL-P was associated with a higher proportion of CD8+CD57+ T-cells. Finally, 1–SD higher medium LDL-P was associated with a lower proportion of Tc2 (CD8+IL-4+), CD8+CD57+, and CD8+CD28–CD57+CD45RA+ (CD45RA+ re-expressing effector memory, TEMRA) T-cells (Figure 2).

### High-density lipoprotein particles (HDL-P)

**Figure 3:**
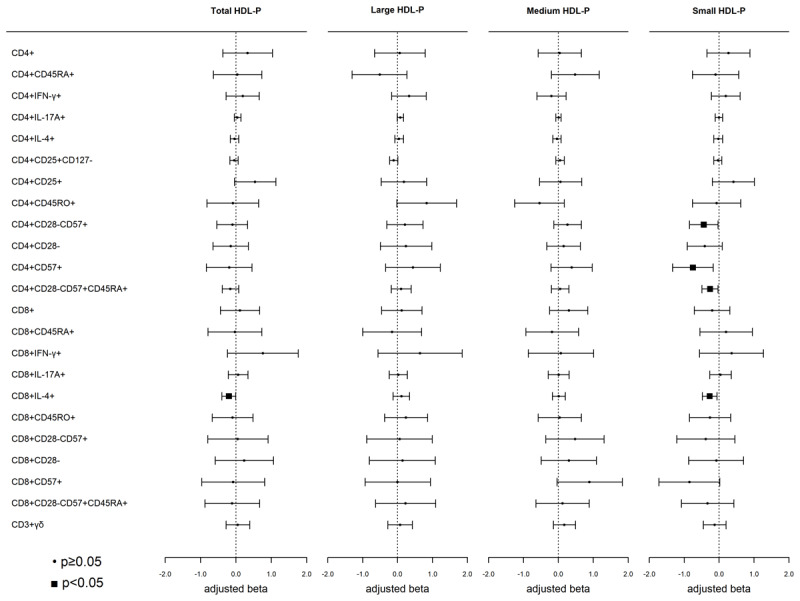
**Associations of high-density lipoprotein particle (HDL-P) fractions with T-cell subsets:** Weighted linear regression models were used with HDL-P as the independent variable and T-cell subsets as the dependent variable. Models were adjusted for age, sex, race/ethnicity, MESA site, cell phenotyping analytical batch, BMI, smoking, hypertensive medications, SBP, diabetes, eGFR, and CMV. Results are presented as beta (95% CI), p-value. Each model represents a single exposure.

Accounting for multiple comparisons, there were no significant associations between HDL-P measures and any T-cell subset (Figure 3). However, a higher concentration (1–SD) of the total HDL-P sub-fraction was nominally associated with a lower proportion of Tc2 T-cells. Additionally, higher small HDL-P was nominally associated with fewer Tc2 and senescent-associated CD4+ T-cells (CD4+CD28–CD57+, CD4+CD57+, and CD4+TEMRA) (Figure 3).

### Triglyceride-rich lipoprotein particles (TRL-P)

**Figure 4:**
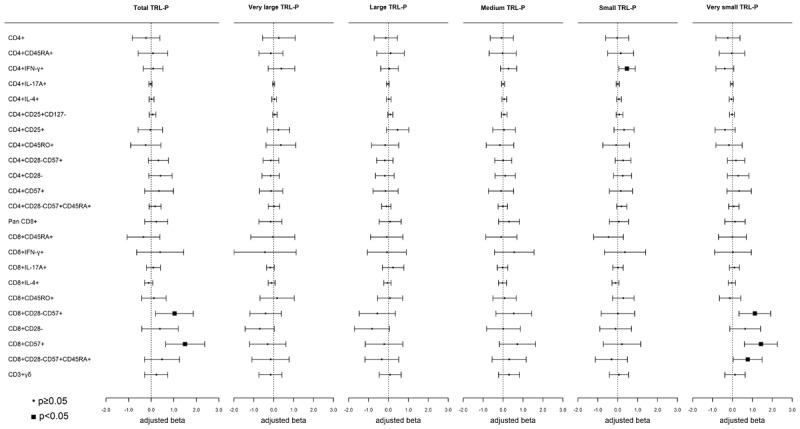
**Associations of triglyceride-rich-lipoprotein-particles (TRL-P) and CD4+ and CD8+ T-cell subsets:** Weighted linear regression models were used with TRL-P as the independent variable and T-cell subsets as the dependent variable. Models were adjusted for age, sex, race/ethnicity, MESA site, cell phenotyping analytical batch, BMI, smoking, hypertensive medications, SBP, diabetes, eGFR, and CMV. Each model represents a single exposure. Results are presented as beta (95% CI), p-value.

There were no significant (p < 0.0006) associations between TRL-P sub-fractions and CD4+ or CD8+ T-cells. However, several nominal associations were observed. Higher concentration (1–SD) of the small TRL-P sub-fraction was nominally associated with a higher proportion of Th1 T-cells (Figure 4). Higher total and very small TRL-P sub-fractions were nominally associated with higher CD8+CD28–CD57+ and CD8+CD57+ T-cells; higher very small TRL-P was also nominally associated with higher CD8+ TEMRA T-cells (Figure 4).

### Associations of Lipids and Lipoprotein Particles with B-cell and Innate Immune Cell Subsets

We also assessed associations between lipid and lipoprotein-particle concentrations with CD19+ B-cell, innate lymphocyte, and CD14+ monocyte subsets in exploratory analyses (Supplementary Tables 2–5). Higher concentration (1–SD) of HDL-C was significantly associated with a higher proportion of CD19+CD27+ (memory) B-cells (β = 1.97% (95% CI: 1.01, 2.94%), p = 0.0001) and higher total-cholesterol was nominally associated with higher memory B-cells (Supplemental Table 2). Additionally, higher concentrations of HDL-C and triglycerides were nominally associated with a lower proportion of pan CD19+ B-cells.

Consistent with HDL-C, higher concentration of total HDL-P was significantly associated with a lower proportion of pan B-cells (β = –0.78% (95% CI: –1.19, –0.38%), p = 0.0002). Further, a 1–SD increment of large and small HDL-P was nominally associated with a lower proportion of pan CD19+ B-cells. Higher total and large HDL-P were also nominally associated with a higher proportion of memory B-cells (Supplemental Table 4).

Higher (1–SD) small HDL-P was nominally associated with a higher percentage of classical monocytes (CD14++CD16–) and higher total HDL-P was nominally associated with fewer natural killer cells (CD3–CD16+CD56+) (Supplemental Table 4). Higher very small TRL-P was nominally associated with a higher percentage of natural killer cells (Supplemental Table 5). There were no associations between LDL-P fractions and these immune cell subsets (Supplementary Table 3).

### Sex and race/Ethnicity interactions

Lastly, we tested lipid/lipoprotein-by-sex and lipid/lipoprotein-by-race/ethnicity interaction terms to assess whether there were differences in the relationships by sex or race/ethnic group. Several LDL-P fractions were inversely associated with senescence-associated CD8+ T-cells in men, but not women. There were also associations of LDL-C and LDL-P fractions with lower CD4+ and CD8+ T-cells expressing markers of inflammation or differentiation in White or Black participants, respectively, but not among the other race/ethnic groups (p-interaction < 0.05; Supplementary Tables 4 and 7). Although several nominally significant associations were observed within these strata, none were significant as defined using the pre-specified significance threshold (p < 0.0006).

## Discussion

We investigated relationships between circulating lipids, lipoprotein particles, and immune cell subsets in the community-based MESA cohort. Following correction for multiple hypothesis testing a higher concentration of plasma HDL-C was significantly associated with a higher percentage of memory B-cells, and higher total HDL-P was significantly associated with a lower proportion of pan CD19+ B-cells. Multiple nominally significant associations were observed between lipid and lipoprotein sub-fractions with effector and senescence-associated CD4+ and CD8+ T-cell subsets. However, because these nominal associations did not remain significant after correcting for the large number of multiple comparisons, any interpretation of such exploratory findings should be approached with caution and interpreted as hypothesis generating.

Hypercholesteremia has been linked with increases in CD4+ TREG and Th17 populations (35) and shifts from Th1 to Th2 T-cells and TREG to Th1 and T-follicular helper T-cells in animal models (36,37). In the current study of a multi-ethnic population free of clinical CVD, we did not observe any significant associations between plasma cholesterol concentration and CD4+ TREG (CD4+CD25+CD127–), Th1 (CD4+IFN-γ+), or Th17 (CD4+IL-17 A+) T-cells. These findings are consistent with results from the MEGA Study, which did not observe associations between total-cholesterol and CD4+ TREG subsets in a German cohort (43). Additionally, we observed a nominal association between higher total cholesterol and memory B-cells (CD19+CD27+), which was not observed in the MEGA Study (43). Previously, experimental studies have shown that higher intracellular T-cell cholesterol influences T-cell senescence and memory T-cell formation, differentiation, and fate (16,17). In the current study we observed nominal associations between higher total-cholesterol and CD8+ senescence-associated T-cells (CD8+CD28–CD57+ and CD8+CD57+) but did not observe an association with plasma total cholesterol and memory T-cells. Although it should be noted that intracellular cholesterol was not measured in our study.

We did not identify any associations between HDL-C and T-cell subsets in our study. Results from a prior MESA study, Copenhagen City Heart Study, UK Biobank, and LifeLines DEEP reported that higher HDL-C was significantly (MESA, Copenhagen City Heart Study, UK Biobank) or nominally associated (LifeLines DEEP) with lower lymphocyte counts (38,39,40,41). The MEGA Study identified associations of HDL-C with higher TREGS (43). We did not observe associations with HDL-C and CD4+ TREGS in the overall MESA cohort but did detect a significant race-by-HDL-C interaction term, with trends for an association of higher HDL-C with lower TREGS in Black participants only. In the overall study sample, higher HDL-C was also significantly associated with higher memory B-cells and nominally associated with lower pan CD19+ B-cells. Moreover, several HDL-P fractions were also associated with higher memory and lower CD19+ B-cells. B-cells possess a membrane HDL receptor (scavenger receptor BI) which regulates cellular uptake of cholesterol esters (52,53,54). Genetic knockout of this receptor in mice increased B-cell proliferation and expansion (54). Thus, the inverse association observed between HDL-P and CD19+ B-cells may be related to lower B-cell proliferation, potentially involving activation of scavenger receptor BI. As we analyzed memory B-cells as a proportion of CD19+ B-cells, a smaller overall parent population of CD19+ B-cells may result in a concomitant elevation in the proportion of CD19+ B-cells expressing CD27+, as opposed to an actual increase in numbers of these cells. However, we did not evaluate cell number in the current study and cannot verify this possibility. The associations between HDL and B-cells observed here are generally consistent with results from the MEGA Study which showed higher HDL-C was associated with lower naive B-cells and nominally associated with higher memory and lower pan CD19^+^ B-cells (43).

Although not surpassing the threshold criteria for multiple hypothesis testing, higher concentration of small HDL-P was nominally associated with lower proportions of several CD4+ T-cell subsets expressing markers associated with repeated antigen stimulation and senescence, including lower CD4+CD57+, CD4+CD28–CD57+, and CD4+ TEMRA T-cells. Consistent with these findings, a UK Biobank study also reported that the primary HDL-P protein (apolipoprotein A-I, apoA-I) was cross-sectionally associated with lower lymphocytes (39). However, contrary to our findings, the LifeLines DEEP study observed higher medium and small HDL-P were associated with higher lymphocyte counts (40). ApoA-I was also associated with TREGs in older (median age 62 years) men and women with coronary artery disease and was found to prevent the conversation of TREGS towards a pro-inflammatory phenotype in mouse models (37). As MESA participants were free from clinical CVD at baseline, this could explain the discrepant results observed here for HDL-P and TREGS with those from Gaddis et al. (37).

In the Duke Established Populations for Epidemiologic Studies of the Elderly (D-EPESE) cohort, small HDL-P was predictive of longevity (55). Small HDL-P was also associated with a lower mortality biomarker (Inflammatory Vulnerability Index, a component of the Metabolic Vulnerability Index) in the Rochester Epidemiology Project, CATHGEN Biorepository, and the Intermountain Heart Collaborative Study (56,57). Thus, it is interesting to speculate that one mechanism through which small HDL-P might influence longevity is through the regulation of senescent-associated T-cells. In vitro evidence suggests HDL-P may reverse characteristics of differentiated and senescent T-cell subsets (9). For example, incubation with HDL-P reversed age-related differences in IL-2 production (9), which is a hallmark of T-cell anergy. T-cells incubated with HDL-P isolated from older adults had reduced proliferation compared with T-cells incubated with HDL-P isolated from young adults (9). Aging has also been associated with ‘poorer quality’ HDL-P (58), due to the production of advanced glycation end products via fructation, fructosylation, or glycation (58,59,60). The fructation or glycation of apoA-I may reverse the anti-inflammatory and anti-senescent characteristics of HDL-P (60,61). Thus, changes in HDL quality or HDL-P size may influence healthy aging by modulating T-cell senescence. However, additional research is required to test this hypothesis and further understand these relationships and mechanisms.

LDL-Ps have been demonstrated to influence T-cell activation and function. For example, modified (e.g., oxidized) and non-modified LDL-Ps were shown to induce T-cell activation and increase effector function via antigen presentation or through interaction with the lipoprotein receptor on the T-cell membrane (20,21,62,63,64,65). Oxidized LDL may also influence the pro-inflammatory fate of naive T-cells, as it was shown to prevent their differentiation into anti-inflammatory TREGS and shifted cell differentiation towards a pro-inflammatory lineage (37,62). In the present study, higher total LDL-P was nominally associated with higher Tc17 T-cells (CD8+IL-17A+), and large LDL-P was nominally associated with higher senescence-associated CD8+CD57+ T-cells. Results from the UK Biobank showed that higher concentration of apoB, a main protein in LDL, was cross-sectionally associated with higher lymphocyte counts, while LDL-C was not (39). Interestingly, both higher LDL-C and apoB concentrations were longitudinally associated with higher lymphocyte counts (39). These findings are somewhat consistent with the results of our study and may indicate that LDL induces T-cell effector differentiation. However, the authors of that study did not measure specific T-cell subsets, and the current study did not evaluate apoB, thus this hypothesis cannot be confirmed. Conversely, higher medium LDL-P was nominally associated with a lower percentage of Tc2 (CD8+IL-2+) and senescence-associated CD8+ (CD8+CD57+ and CD8+TEMRA) T-cells. A previous study demonstrated that oxidized-LDL shifted naive T-cells towards pro-inflammatory Th1 T-cells and reduced Th2 activity (62). Smaller LDL-P subclasses are more susceptible to oxidation (66). Thus, although we did not measure modified LDL-P, it is possible that oxidized small LDL-P sub-fractions may have contributed to the association with effector and senescent-associated T-cell subsets. Because LDL has been shown to activate T-cells, it may be possible to leverage this response and immunize against lipoproteins to generate clinically beneficial cell-mediated immunity. Indeed, pre-clinical studies have shown immunization against various lipoproteins for seven weeks protected against atherosclerosis in response to a 17-week high-cholesterol diet (67). Similarly, immunization of CD4^+^ T-cells reactive to LDL protected against atherosclerosis and reduced plasma lipids in humanized mice, likely by activating antibody producing B-cells (21). Whether the protective effect of lipoprotein immunization is due to a humoral response is debated, however (67). Given the link between T-cells and atherosclerosis pathogenesis (68,69,70), including our observations that several T-cell subsets were associated with atherosclerosis markers in MESA (71,72), we hypothesize that immunization against LDL may serve as novel therapy to prevent, delay, or reverse atherosclerosis and downstream CVD risk.

Higher blood triglycerides and small TRL-P were nominally associated with higher Th1 T-cells and total and very small TRL-P sub-fractions were nominally associated with higher CD8+CD28–CD57+, CD8+CD57+, or CD8+TEMRA T-cells. Plasma triglycerides were also nominally associated with fewer CD19+ B-cells. Interestingly, we also observed a sex interaction on the association of triglycerides with a higher concentration associated with lower memory B cell proportions in men, but higher proportions in women. These results are generally consistent with previous findings from UK Biobank and LifeLines DEEP Study, which observed that higher plasma triglyceride concentrations were associated with higher lymphocyte counts (39,40). TRL-P are considered risk factors for inflammation and atherosclerotic cardiovascular disease (73), and are thought to be primarily comprised of chylomicrons and very low-density lipoprotein particles [VLDL-P (73,74)]. Collectively, these results may suggest that TRL-P sub-fractions exert inflammatory effects via VLDL-P induced T-cell activation and, eventually, the expression of a ‘senescent-like’ secretory phenotype. Indeed, in vitro treatment of CD3+ T-cells with VLDL-P blunted T-cell proliferation, activation, and cell cycle progression (75), which are characteristics of senescent T-cells. The same effects, however, were not observed following treatment with LDL-P (75). Consistent with this hypothesis, the LifeLines DEEP Study observed that concentrations of various VLDL-P subclasses were associated with higher lymphocytes (40). However, more research is needed to differentiate the effect of triglycerides and TRL-P, LDL-P, and VLDL-P sub-fractions on T-cell effector function and senescence.

Finally, we observed interaction effects of sex and race/ethnicity on the associations between additional plasma lipids and lipoproteins and several circulating T-cell subsets. Although several nominally significant associations were observed within these strata, none were significant as defined using the pre-specified significance threshold (p < 0.0006). In particular, various LDL-P fractions were nominally associated with senescence-associated CD8+ subsets in men but not women. LDL-P fractions were nominally associated with higher Th1 T-cells in men and LDL-C concentration with lower Th17 T-cells in women. LDL-C and LDL-P concentrations were also nominally associated with lower CD8+CD45RA+ and Tc1 T-cells in Black participants, respectively, and medium LDL-P was nominally associated with lower CD4+CD45RO+ T-cells in White participants with no relationships in other race/ethnic groups. The reasons for an association of lipoproteins that increase the risk for CVD with lower senescent- or inflammation-associated CD8+ subsets are not apparent. MESA participants were free of clinical CVD as an enrollment criterion at the baseline exam. It is possible relationships of LDL with T cell subsets observed here would change in participants with clinical CVD. Somewhat consistent with our findings, the UK Biobank reported several sex interactions for the relationships between LDL-C, HDL-C, triglycerides, apoA-I, and Lp(a) with lymphocytes (39). Although our analyses of specific lymphocyte subsets make comparisons difficult, a prior study of lipids and leukocyte population counts in MESA observed no interaction effects of sex or race/ethnicity aside from a race/ethnicity interaction for the association between triglyceride concentration and total leukocytes (38). Additionally, NHANES also showed sex differences in relationships of blood lipids with multiple leukocyte population counts (42). However, despite these findings we urge caution when interpreting our stratified results due to the large number of statistical tests that were performed. These nominally significant results did not surpass the significance threshold for multiple hypothesis testing, are highly exploratory, and prone to type I error. We suggest interpreting these results cautiously as they substantially increase the number of tests performed and contain small sample sizes in some of the strata. Further replication of these findings are needed.

Several additional study limitations are noted. First, the data are observational and cross-sectional, and we cannot infer temporality, causality, or draw inferences on potential molecular mechanisms. Second, both the lipoprotein and cell data were measured at a single time point, and we do not know the length of time the peripheral immune cells were exposed to lipid fractions or lipoprotein particles. Third, the analyses estimated associations of plasma concentrations of blood lipoprotein and cholesterol particles, did not study relationships of intracellular cholesterol with T-cell differentiation or function, and did not assess relationships with modified species (e.g. oxidized LDL), or other lipoproteins that are important CVD risk factors (i.e., apoB and lipoprotein(a)). These are major limitations, given the large body of literature demonstrating the influence of intracellular cholesterol (14,15,16,17,24,25,26,27,28,29,30,31,32,33,34) and oxidized LDL (21,37,62,63,64,76) on T-cell function and differentiation. Fourth, the phenotyping of immune cell diversity was limited, and we may have missed associations as a result. Fifth, because the peripheral immune cells were phenotyped using flow cytometry for surface proteins and intracellular cytokines, we do not have transcriptional, proteomic, metabolomics, or other functional data. Sixth, because MESA participants were free from clinically CVD at baseline, the results may not be generalized to clinical populations. We also did not adjust for other potentially relevant confounders such as physical activity, diet, sleep, or inflammatory biomarkers. Further research is needed to understand the influence of these and other potential confounders on the associations between lipids and lipoproteins and the immune system. Seventh, and most importantly, the majority of findings did not reach the pre-determined statistical significance threshold that corrected for multiple testing. We consider all nominally significant findings to be exploratory for the purposes of hypothesis generation and urge caution in interpreting these results. While the nominally significant findings may have plausible biological mechanisms, there is a risk such associations are spurious. Independent replication is required to confirm their validity.

The strengths of this study include evaluation of several lipoprotein particle subclasses evaluated by NMR and a large panel of immune cell phenotypes assayed among a community-based cohort. We interpret the overall findings from this study as hypothesis-generating, requiring confirmation in future longitudinal studies with serial measures of lipids and immune cells. Long-term clinical trials using FDA approved lipid-lowering medications (e.g., statins) are also warranted to understand the effect of lipids on the immune system, and several recent, short-term clinical trials have already been performed (77,78,79,80). Additionally, in vitro and animal studies are required to elucidate the mechanisms through which lipoproteins and their lipid cargo affect T-cells and the immune response. This may help determine the potential of novel therapeutics targeting lipid-immune interactions in clinical settings.

In summary, higher concentrations of blood HDL-C and total HDL-P were significantly associated with higher proportions of CD19+ memory B-cells and lower proportions of pan CD19+ B-cells, respectively. Multiple blood lipid and lipoprotein sub-fractions were associated with CD4+ and CD8+ T-cell subsets characterized by markers associated with effector and senescence-associated subsets. These results suggest chronic exposure to high total cholesterol, triglycerides, LDL-C, TRL-P, and LDL-P may influence T-cell differentiation, proliferation, and cell cycle function, while higher HDL-C and HDL-P may act in a counter fashion. These findings, however, need to be replicated in other cohorts and in longitudinal studies, and should be interpreted cautiously given the cross-sectional nature, nominally significant and exploratory findings, and single cohort design. If corroborated, these findings might have implications for the development of therapeutics targeting inflammation and cardio-metabolic diseases in patients with elevated lipids.

## Data Accessibility Statement

MESA data can be requested from the Collaborative Health Studies Coordinating Center (CHSCC) upon approval of a paper proposal using this data. The instructions are at https://www.mesa-nhlbi.org/. MESA data is also available via BIOLINCC (https://biolincc.nhlbi.nih.gov/home/, dbGaP Study Accession: phs003288.v1.p1). The corresponding author can be contacted for the analytic methods. Any questions or interest in the immune cell data, lipid or lipoprotein particle data, or other study materials should be directed to MESA via the study authors or the CHSCC.

## Supplemental Material

Supplemental materials can be accessed at https://figshare.com/s/ef0888b39d0e18e82374.
